# Modular Software for Generating and Modeling Diverse Polymer Databases

**DOI:** 10.1021/acs.jcim.3c00081

**Published:** 2023-06-08

**Authors:** Alejandro Santana-Bonilla, Raquel López-Ríos de Castro, Peike Sun, Robert M. Ziolek, Christian D. Lorenz

**Affiliations:** † Department of Physics, King’s College London, London WC2R 2LS, United Kingdom; ‡ Department of Chemistry, King’s College London, London SE1 1DB, United Kingdom; § Biological Physics and Soft Matter Group, Department of Physics, King’s College London, London WC2R 2LS, United Kingdom

## Abstract

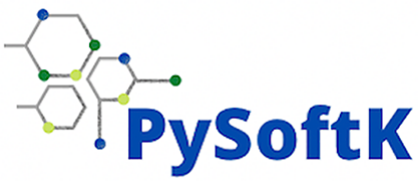

Machine learning methods offer the opportunity to design new functional materials on an unprecedented scale; however, building the large, diverse databases of molecules on which to train such methods remains a daunting task. Automated computational chemistry modeling workflows are therefore becoming essential tools in this data-driven hunt for new materials with novel properties, since they offer a means by which to create and curate molecular databases without requiring significant levels of user input. This ensures that well-founded concerns regarding data provenance, reproducibility, and replicability are mitigated. We have developed a versatile and flexible software package, PySoftK (Python Soft Matter at King’s College London) that provides flexible, automated computational workflows to create, model, and curate libraries of polymers with minimal user intervention. PySoftK is available as an efficient, fully tested, and easily installable Python package. Key features of the software include the wide range of different polymer topologies that can be automatically generated and its fully parallelized library generation tools. It is anticipated that PySoftK will support the generation, modeling, and curation of large polymer libraries to support functional materials discovery in the nanotechnology and biotechnology arenas.

## Introduction

The diverse chemical, mechanical, and electronic properties of polymers underlie their application in relevant technological areas spanning structural materials, cosmetics, pharmaceutical formulation, electronics, and biotechnology.^1−10^ In order to optimize their aforementioned properties for this range of applications, the roles of polymer constitution and topology have been widely explored, with a range of architectures including homopolymers, block copolymers, and branched and ring polymers.^11−17^ As a result of contemporary research activities, the chemical and structural domains of synthetic polymers are continuously growing.^18−20^

Polymeric materials have been the focus of a significant amount of scientific research for many decades. In more recent years, access to ever-growing computational power has allowed the materials modeling community to carry out increasingly complex investigations of polymeric materials.^21−28^ Modern computer simulation studies provide predictive understanding of molecular interactions and mechanisms that determine the bulk-scale properties of polymers of interest. In tandem with experimental data, this combined insight yields rational design principles for new polymers with enhanced target properties.

While there are well-established computer simulation techniques to support the investigation of polymers, often the most technically difficult part of a polymer simulation workflow is actually generating models of the polymer(s) of interest. As a result, multiple computational platforms have been developed in recent years with the aim of making the generation of polymer models more straightforward.^29−33^ Several of these packages have been developed to allow users to build molecular models of polymers for use in molecular dynamics simulations. These packages generally require the user to provide a detailed description of the underlying chemistry of, and connectivity between, constituent monomers to output a topology of the desired polymer. Some allow the user to input force field information to be used in generating input files for classical molecular dynamics simulations. All of the referenced codes allow for the user to build homopolymers while some allow the user to build heteropolymers with different architectures and topologies.^29−32^

More recently, the Polymer Structure Predictor (PSP)^34^ has reduced the amount of information required from the user to describe the chemistry of the monomers by allowing a SMILES string to be used as the input. The code can assign force field parameters from the CHARMM, AMBER, and OPLS generalized force fields^35−37^ and integrates with the LAMMPS classical simulation engine^38^ and the pysimm package^29^ for optimization of polymer structures. While the amount of the predefined information required to build the polymers is less in PSP than is required by other packages, the polymers that can currently be built are limited to homopolymers with either linear or cyclic topologies.

Here, we present PySoftK, a modular and versatile code to model polymer structures with different topologies. We present the various modules that currently exist within PySoftK to build different polymer structures and show how they can be combined in order to build highly complex polymer topologies in an automated way. We subsequently demonstrate the various functionalities that are found within the code to facilitate high-throughput molecular modeling calculations. PySoftK can also be used in the parametrization of dihedral terms in conjugated polymers, which are commonly poorly defined by standard classical force fields.^28,39^ Finally, we briefly review the steps that have been taken when developing the code to ensure it can be successfully used in a broad range of applications.

## Software Overview

PySoftK is a modular Python package that generates molecular models of polymers with a diverse range of topologies and chemistries with minimal input from the user. It also has various tools to facilitate high-throughput simulations of polymers. The code utilizes RDKit to generate the molecular models of the various polymers.^40^ The modular nature of this package will allow the scientific community to easily expand the code base to include polymer architectures that are not included in this first release of the library.

### Generating Diverse Polymer Architectures

#### Defining Monomers

**Figure 1:**
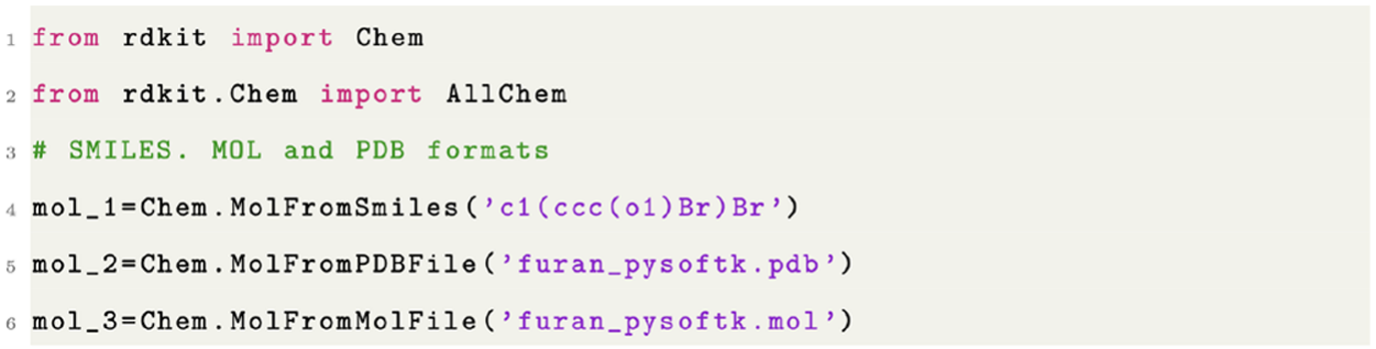
Code snippet showing the different input formats that PySoftK accepts.

**Figure 2:**
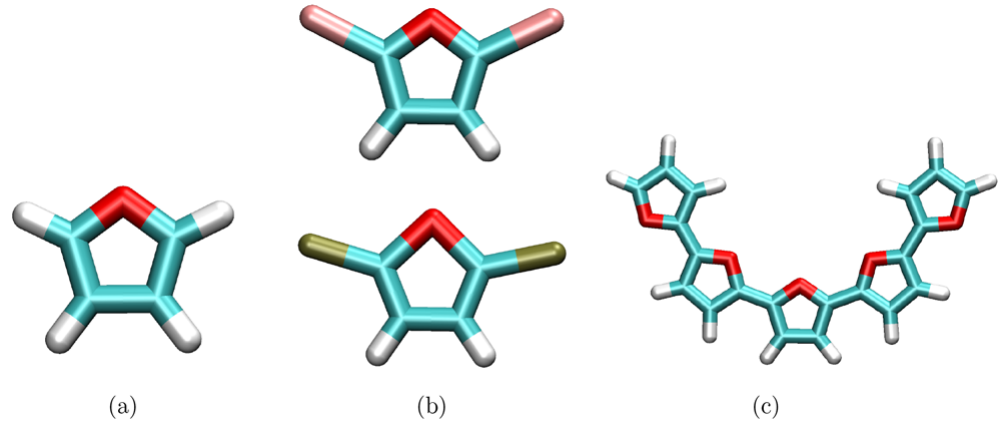
Creation of a linear polymer. (a) Undecorated single furan molecule. (b) Decorated furan molecule (using a Br or Pt atom) highlighting the region where intermolecular bonds are formed between monomers. (c) Linear polymer with a five monomers and optimized atomic positions at the RDKit **MMFF** force-field level.

A key functionality of PySoftK is the automated generation of a diverse range of polymer architectures. The user-inputted monomers, in which the sites that link each monomer together are predefined, are the main topological descriptor used by PySoftK. The chemical structure and connectivity of the monomer(s) that make up the polymer can be inputted using all formats supported by RDKit, such as SMILES strings and .pdb, .mol, and .xyz files, as shown in Figure 1. Examples of inputs to input a furan monomer are shown in Figure 2a. In order to construct a polymer from its constituent monomers, a placeholder atom is used to indicate where the bond formation takes place during polymerization. In the examples presented in this paper, bromine (Br) atoms or platinum (Pt) are used as these placeholder atoms (see Figure 2b). It is important to note that this choice is entirely arbitrary: any atom can be used as the placeholder atom. The placeholder atoms are used only to build a given polymer; once the construction is complete, the remaining placeholder atoms are replaced by hydrogen atoms.

#### Generating Polymers with Different Monomer Distributions

Once the monomers are defined, then PySoftK can be used to describe the distribution of the monomers within the polymer and the overall architecture of the polymer. The code is structured such that specific modules are provided to generate homopolymers, diblock copolymers, and random polymers. Random polymers are simply built such that the different types of monomers are distributed randomly within the polymer. Block and random copolymers with more than two types of monomers, as well as alternating, periodic, and statistical copolymers of any number of monomers, can be built using the patterned module within PySoftK. The modular structure of the software allows users to develop their own codes to design additional polymer topologies. In the following sections, examples of the different modules to build the various polymer topologies with different distributions of the monomers are provided.

#### Generating Polymers with Different Topologies

Users can generate models of polymers with three different general topologies: (i) linear, (ii) cyclic, and (iii) branched. In the following sections, examples showing how to use the various modules to build each block and random copolymers with each topology are presented. Finally, we present the patterned module, which provides the user more flexibility in defining the distribution of the monomers within the polymer and can be used in combination with the various topology modules to build any of these different polymer architectures.

#### Linear Polymers

**Figure 3:**
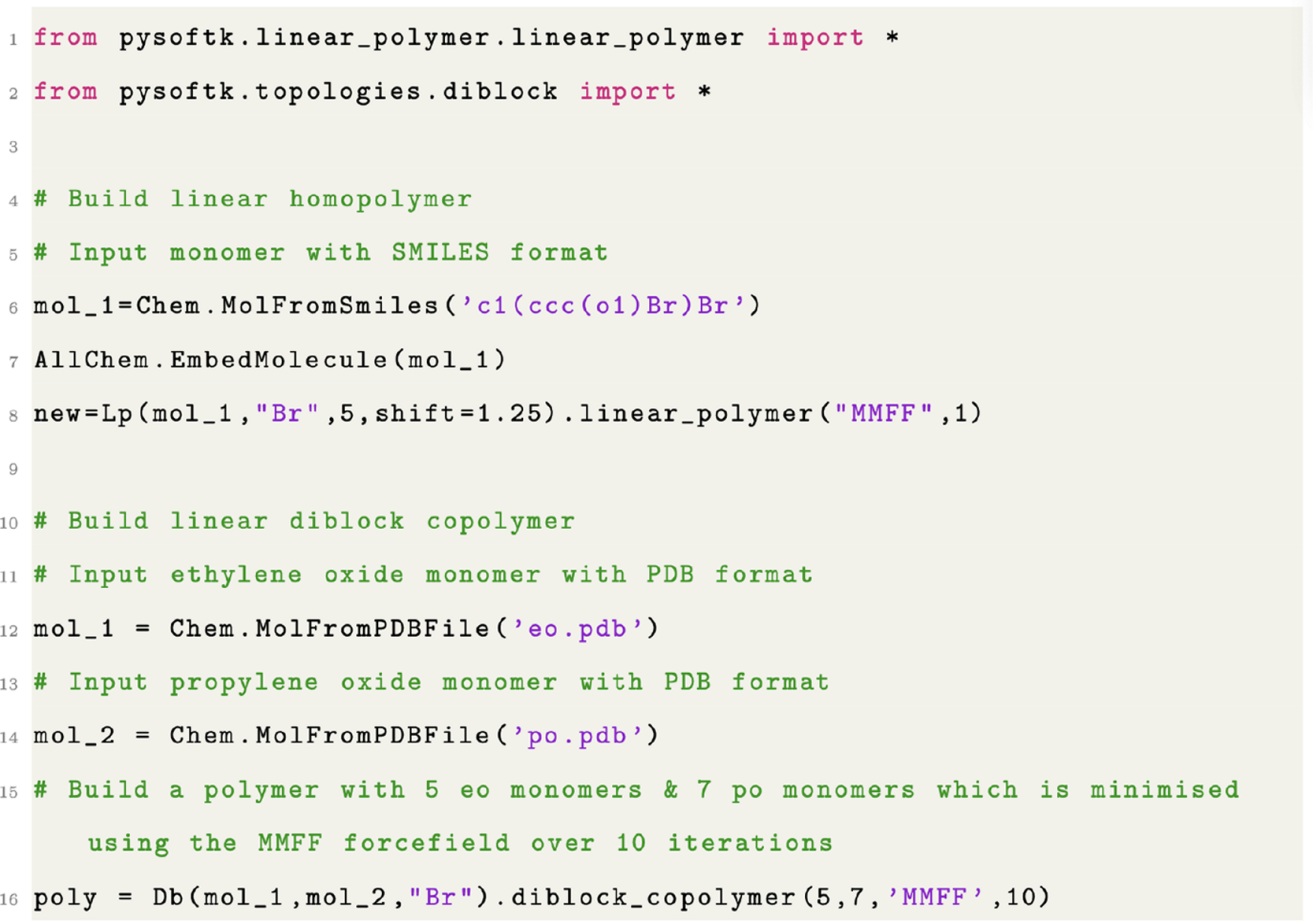
Code snippet showing the creation of a linear homopolymer and a linear diblock copolymer.

Linear polymers consist of individual monomers which are joined together end-to-end forming a single molecule. In order to build a linear homopolymer in PySoftK, the command Lp(mol,atom,n_copies,shift).linear_polymer(FF,iter_ff) is used to initiate a two-step process. First, a monomer (defined by the variable mol) is copied and translated along a predefined axis, where the distance between each monomer is defined by the shift variable in the module call, which results in an unconnected chain of n_copies monomers. Subsequently, a merging step between end-to-end monomers is performed employing a user-designated atomic placeholder (defined by the variable atom) indicating to PySoftK the selected site on the monomer where a bond is formed. Finally, to provide a realistic initial structure, PySoftK utilizes the **MMFF** or **UFF** force-field parametrizations (as chosen with the FF parameter) as implemented in RDKit^40^ in order to generate an energy minimized molecular conformation after iter_ff steps of minimization. This process is displayed in Figure 2, where a single furan molecule is decorated with a placeholder atom (in this case bromine) shown in pink and extended to form a linear polymer containing five monomers. The full block of code required to generate this homopolymer is found in Figure 3.

The Db module within PySoftK allows users to build linear diblock copolymers. The command Db(ma,mb,atom).diblock_copolymer(len_block_A,len_block_B,FF,iter_ff) is used to generate a diblock copolymer that consists of a block containing len_block_A monomers of ma and a block containing len_block_B monomers of mb. Again, the monomers are inputted with atomic placeholders (atom) that identify the polymerization site on each monomer, and the resultant structure undergoes iter_ff steps of energy minimization using the FF force field. Figure 3 shows an example of how to practically apply this function.

**Figure 4:**
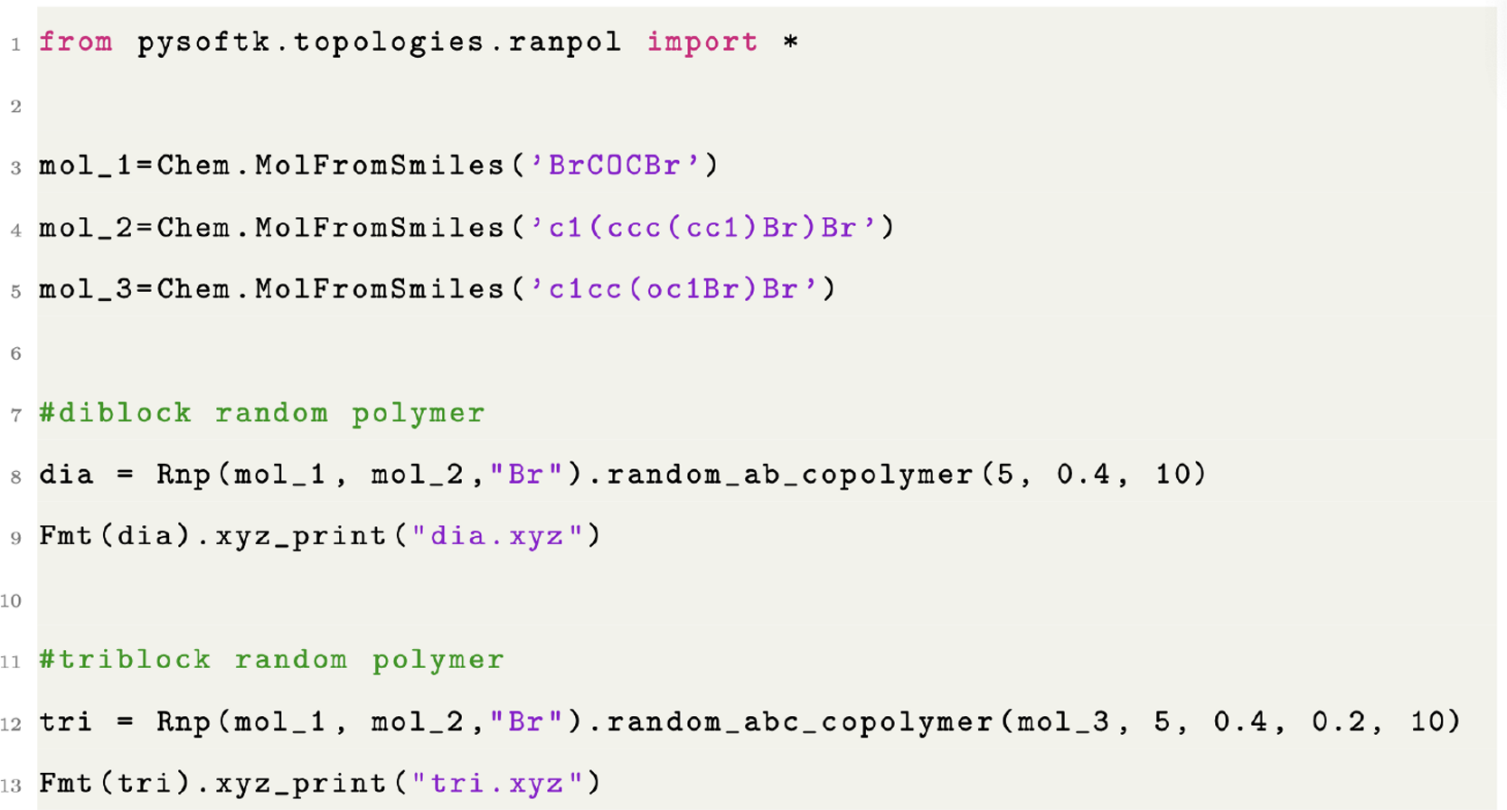
Code snippet showing the usage of the random copolymer module functions. The functions random_ab_copolymer and random_abc_copolymer allow the definition of the user-supplied linking probabilities.

**Figure 5:**
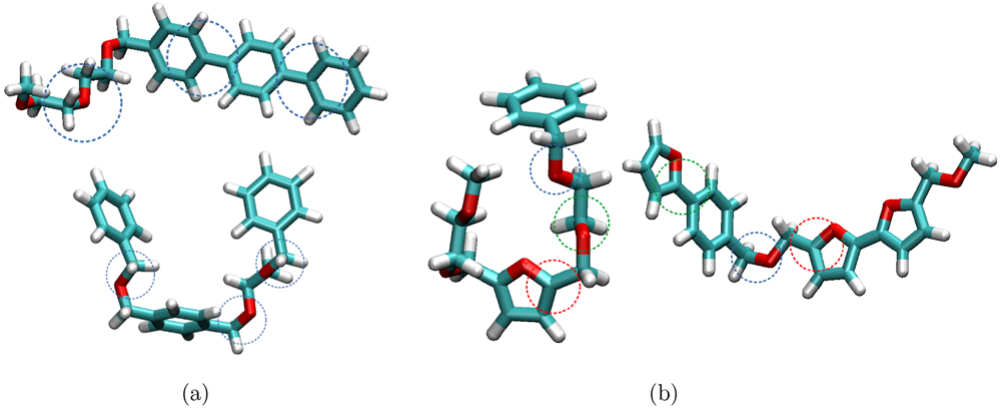
Creation of a random polymer with user defined probabilities. (a) Diblock copolymer architecture, where a single probability (*P*_1_) of being linked is 0.4. The linkages are highlighted with a blue circle. Two different polymeric topologies are obtained using the same probability. (b) Triblock copolymer architecture employing user-defined probabilities *P*_1_ (represented in green) and *P*_2_ (displayed in red). Two different moieties are obtained based on user defined probabilities (0.4 and 0.2), respectively.

Additionally, PySoftK supports the construction of random linear copolymers which consist of two or three different monomers. The module Rnp(mol_1,mol_2,atom).random_ab_copolymer(len,pA,iter_ff,FF) is used to build a random copolymer of length len that contains two monomers, mol_1 and mol_2 which are decorated with atoms of type atom to indicate where the polymerization occurs. The number of mol_1 and mol_2 monomers in the resulting polymer are determined from pA*len and (1-pA)*len, respectively. Similarly PySoftK can be used to generate random linear copolymers consisting of three different monomers via the module Rnp(mol_1,mol_2,atom).random_abc_copolymer(mol_3,len,pA,pB,iter_ff,FF). In this case, the resulting polymer will contain len monomers, such that the numbers of mol_1, mol_2, and mol_3 monomers are pA*len, pB*len, and (1-pA-pB)*len, respectively. A snippet showing the usage of the functions is displayed in Figure 4, and examples of the polymers produced by that code are presented in Figure 5.

#### Ring Polymers

**Figure 6:**
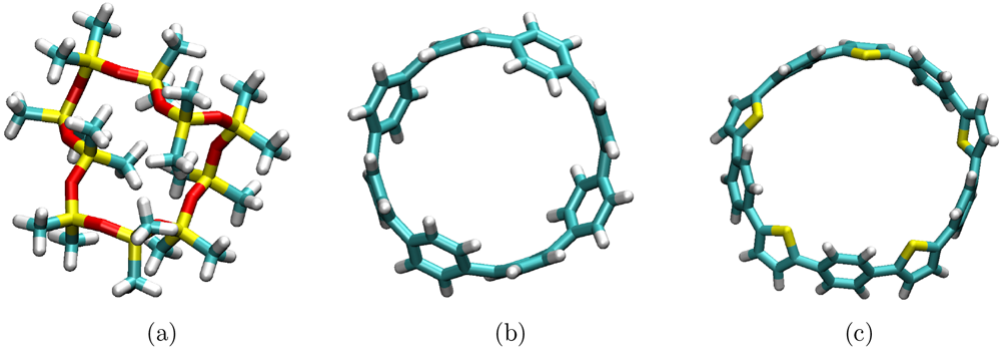
Creation of ring polymers using (a) silanol and (b) benzene ring. (c) Alternating copolymer (benzene and thiol monomers combined via the Sm module). This example illustrates the capabilities of PySoftK to utilize previously developed algorithms for creating new architectures.

**Figure 7:**
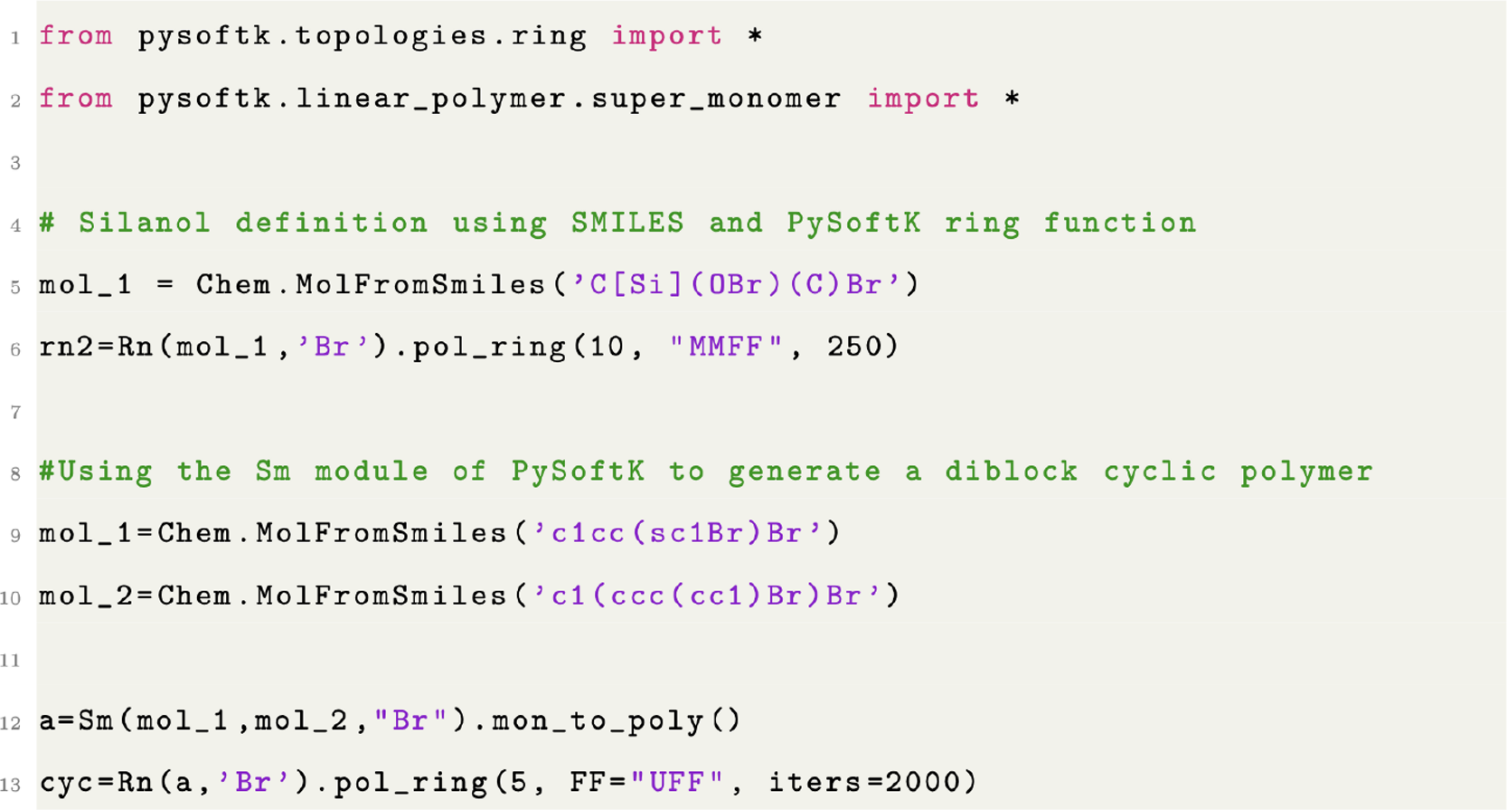
Code snippet showing the creation of a cyclic polymer.

Current experimental techniques allow precise control of polymer architectures enabling the creation of new topologies. One interesting example is ring, or cyclic, polymers, which can be described as closed macromolecular structures with no beginning or end.^41^ PySoftK enables the creation of ring homopolymers with the module Rn(mol_1,atom).pol_ring(len,FF,iter_ff), which generates a ring polymer with len(mol_1) monomers by first employing the linear polymer module Lp to generate a polymer chain. Then, the initial and final atomic placeholders (atom) are used to create a bond, which generates a ring topology as shown in Figure 6(a, b). A snippet of code utilizing this command is shown in Figure 7. It is worth mentioning that PySoftK enables the combination of different modules to create vast numbers of new structures. This is demonstrated in the bottom part of Figure 7, where the Sm module is used to create a linear diblock polymer that is then converted to a ring polymer topology using the Rn module, with the resultant ring polymer shown in Figure 6(c).

#### Branched Polymers

**Figure 8:**
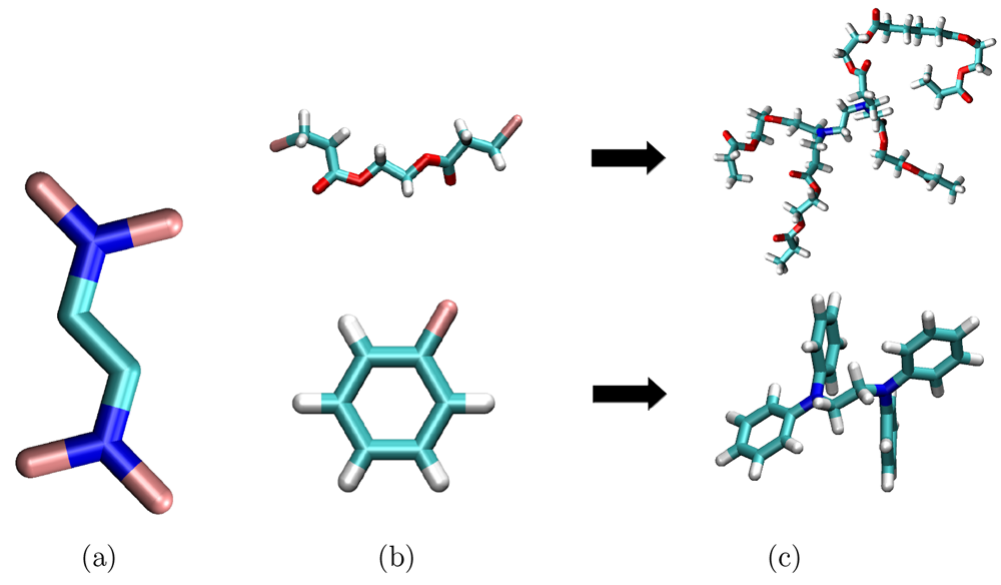
Creation of branched polymers using (a) ethylenediamine as the primary backbone and (b) benzene or polyester oligomer as branches. (c) Branched polymer with provided core and arms moieties. In this case, only one placeholder atom is used to signal PySoftK the region where a bond would be formed.

**Figure 9:**
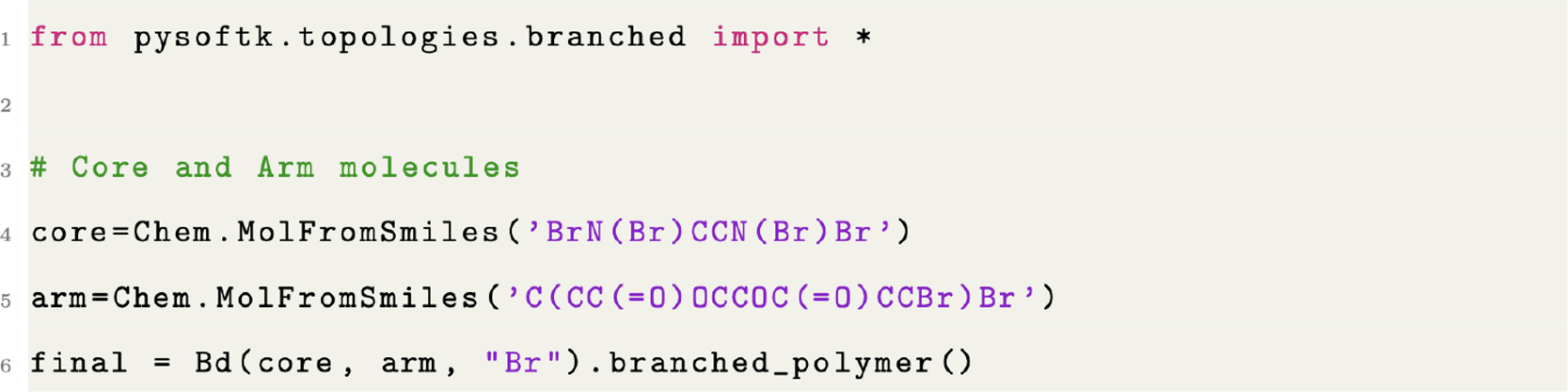
Code snippet showing the creation of a branched polymer.

Branched polymers are another topology that has been enabled in PySoftK. Generally, branched polymers are a set of secondary polymer chains linked to a primary backbone. PySoftK builds this topology by employing the user-supplied atomic placeholder as an indicator of the number of branch points from the primary backbone present in this structure. Thus, for instance, a backbone with four placeholders will generate a model where four branches (arms) are attached at the regions on the backbone indicated by the user, as shown in Figure 8. In PySoftK, the module Bd(core,arm,atom).branched_polymer(FF,ff_iter) is used to generate a branched polymer with a backbone of core and arms described by arm (Figure 9). The placeholder atoms are defined by atom. The inputted structures for core and arm can be simple monomers and therefore inputted as previously discussed, or they can be resultant structures from any of the other commands and then inputted into the branched function. Therefore, the branched architecture not only illustrates the capabilities of PySoftK to create new architectures from scratch but also the versatility of PySoftK to be extended. In this case, this has been done by creating the module topologies where all the previous functions have been organized.

#### Specifying Absolute Monomer Sequences

**Figure 10:**
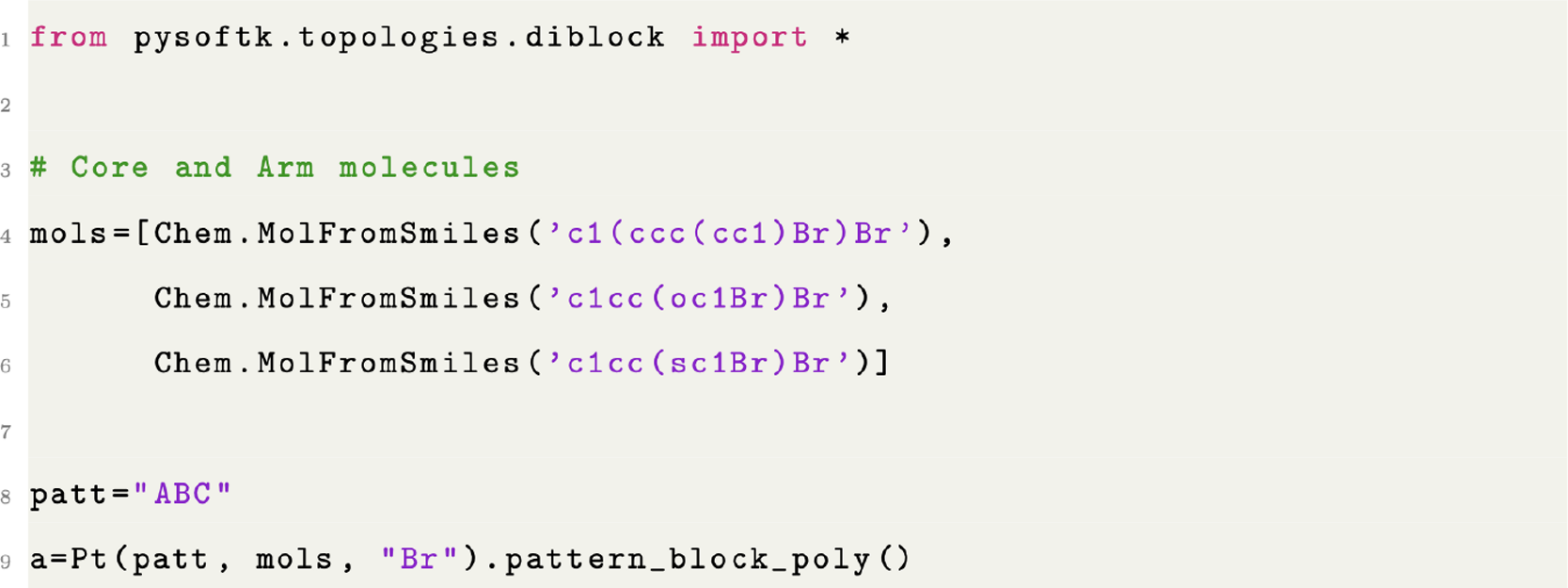
Code snippet showing the creation of a patterned polymer.

**Figure 11:**
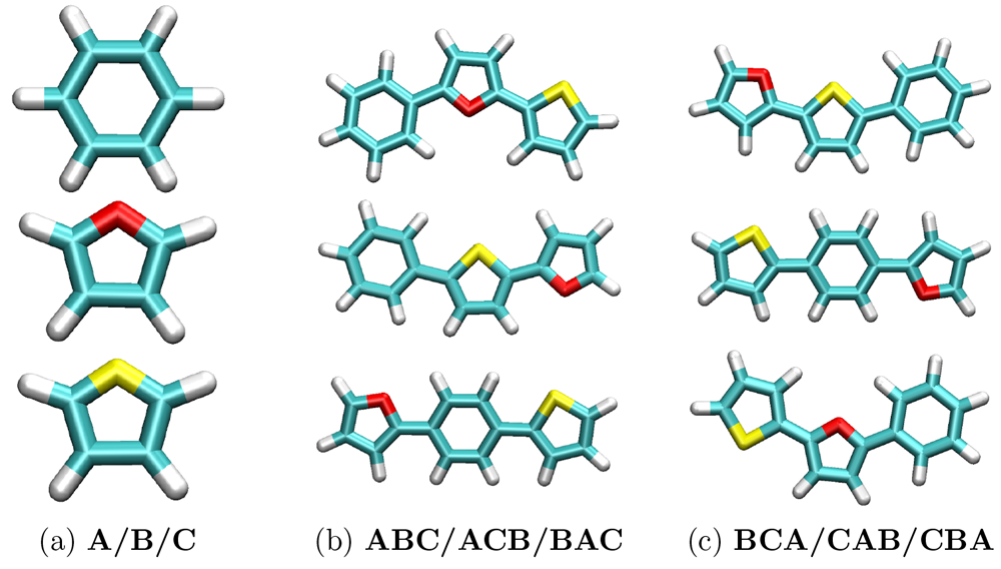
Patterned construction of a polymer based on a user-defined pattern. (a) A user-created list is mapped to an alphabetic string representing the position of the molecule in the list. (b, c) Possible unique permutations for an arbitrary list containing three elements.

Any linear polymer can be described by listing the combination of monomers in a specific pattern (e.g., ABBACCABBACC, ABCABABCBA). Therefore, PySoftK offers the option to build polymers with a specific pattern of monomers using an alphabet-based pattern expressed in a single string followed by a list of RDKit^40^ molecular objects as presented in Figure 10. In Figure 11, thiophene, furan, and benzene monomers are used to present the different possibilities provided by this function to construct all unique permutations.

This function can also be used in conjunction with the linear_polymer module of PySoftK to construct polymeric macromolecules that have other polymers as their monomeric units.

### Facilitating High-Throughput Calculations

#### Folder Creation and Organization

**Figure 12:**
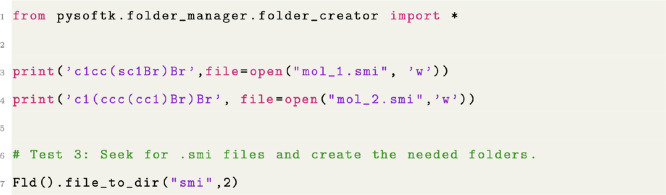
Code snippet showing the automatic creation of a folder to organize files based on an user-defined extension.

The module pysoftk.folder_manager.folder_creator has been designed to create a user-defined number of folders with unique names. In the case of high-throughput calculations (HTC), usually many different systems are created in a single folder and then relocated to enable calculations or postprocessing analyses to be conducted. As shown in Figure 12, this workflow can be carried out utilizing the function Fld().file_to_dir implemented in PySoftK. The result of the code in Figure 12 is that two new, uniquely named directories would be created, and each of the two .smi files (mol_1.smi and mol_2.smi) would be moved into one of these new directories, such that each new directory would contain one .smi file.

**Figure 13:**
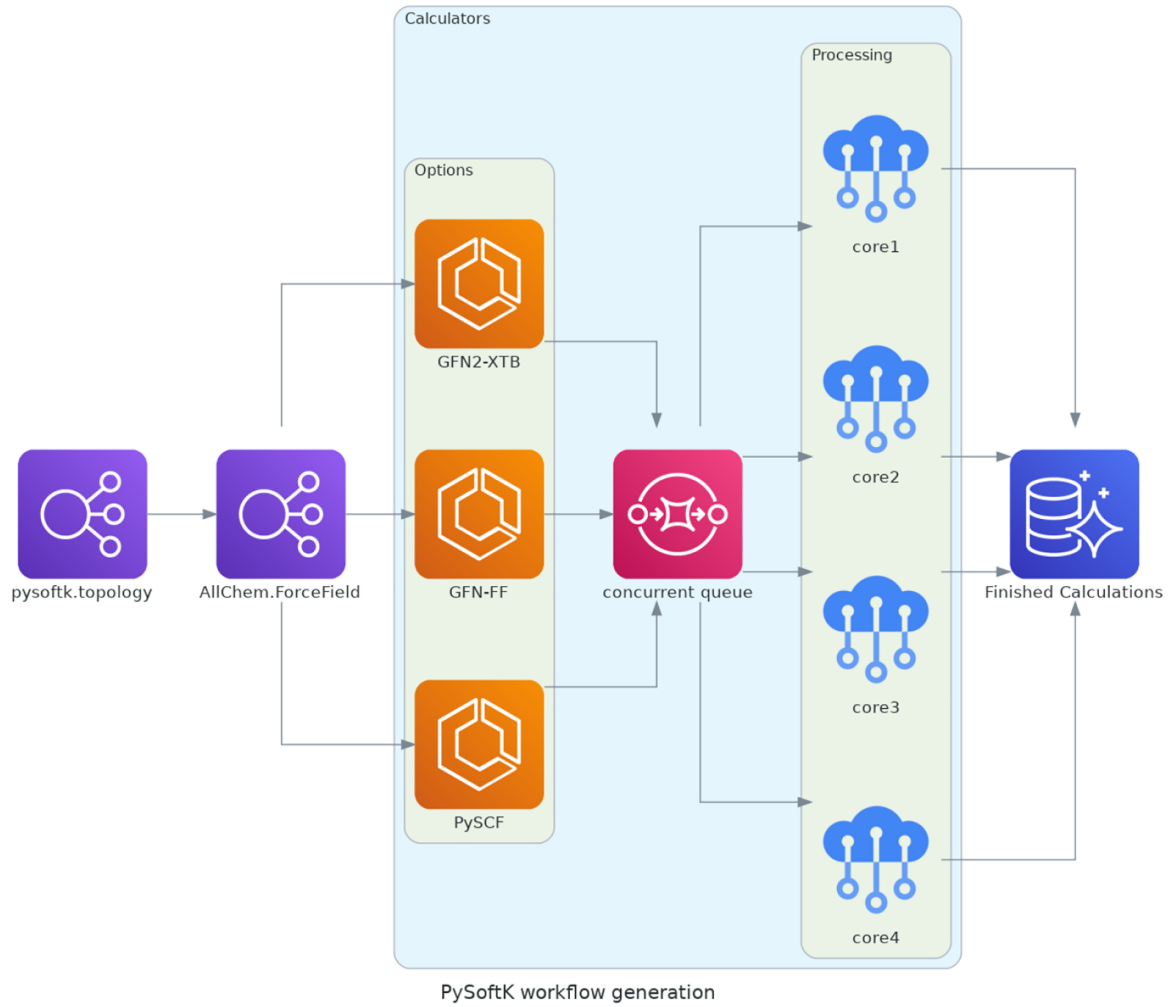
PySoftK workflow generation. Topology objects (polymers) are created and automatically parsed to RDKit force-field optimization. External programs such as GFN2-XTB, GFN-FF, or PySCF are linked using internal modules which facilitates the automation process. Concurrent parallelization allow the creation of events in which subsequent parallelization strategies can be used. In this case, one thread event is controlling a process employing four cores. File organization is performed.

The aforementioned function is able to search for files with a given file extension and relocate them within individually created folders. Likewise, for cases where many files are present, this command can be executed in parallel. The creation of automated workflows (as displayed in Figure 13) can be achieved by combining many different functions in PySoftK enabling the modeling of thousands of polymers using a single script. An example demonstrating how this can be done can be found in the SI. Alternatively, these modules can be used separately as part of another workflow that generates other types of directory structures or simply a folder where many different files are located.

#### Automatic Torsional Angle Detection for Conjugated Polymers

PySoftK offers a tool to perform analysis of the torsion angles within conjugated polymers that connect the ring subunits that are commonly found in their backbone. These dihedrals are generally poorly captured by the existing parameters within classical force fields.^28,39^ Thus, having the capability to rapidly identify those dihedrals then allows the user to easily set up the necessary *ab initio* simulations required to determine the potential energy landscape of those dihedrals, which then can be used to reparameterize the force field for those dihedrals.

**Figure 14:**
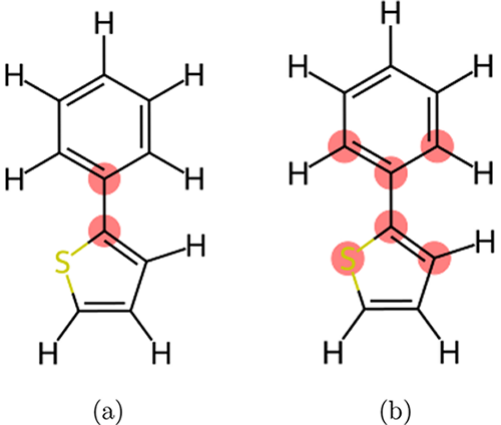
Graphical explanation of the **P-Graph** description and algorithm used for automatically detecting intermolecular torsional angles. (a) Bond linking atoms are detected. (b) All connecting paths are computed where previously detected atoms are used as starting and end points.

The module pysoftk.torsional automatically detects and reports the atoms involved in the torsional angles found within planar conjugated polymers. PySoftK also enables the creation of molecular sketches highlighting and reporting the atom numbers that form a torsional angle as shown in Figure 14. The topological fingerprint used in this module relies on the description of molecules as graphs where the atoms are nodes and the covalent bonds are the edges.^42^ Based on the idea to convert a molecular Graph (**M-Graph**) into a path Graph (**P-Graph**), we have been able to label connections between edges in the **M-Graph** providing a nomenclature to name the **P-Graph**.^43^

In order to identify the torsions between ring subunits within the backbone of a conjugated polymer, we have identified two conditions that the atoms (e.g., nodes) making up the central bond of the torsion must meet. First, an atom must have three neighbors which are from the inner structure (such as rings) and one provided from the next monomer (as displayed in Figure 14a). However, this condition can be also be found in atoms that are embedded within aromatic rings. To avoid this, we have used the ring detection function provided by RDKit to remove potential bonds within a ring and therefore identifty the bonds which connect the ring moieties in the polymer (Figure 14a). After identifying all of these important bonds within the polymer, we then identify all of the torsions that include these bonds as the central bond and therefore can provide the relevant atomic labels involved in these torsional angles as shown in Figure 14b.^44^

**Figure 15:**
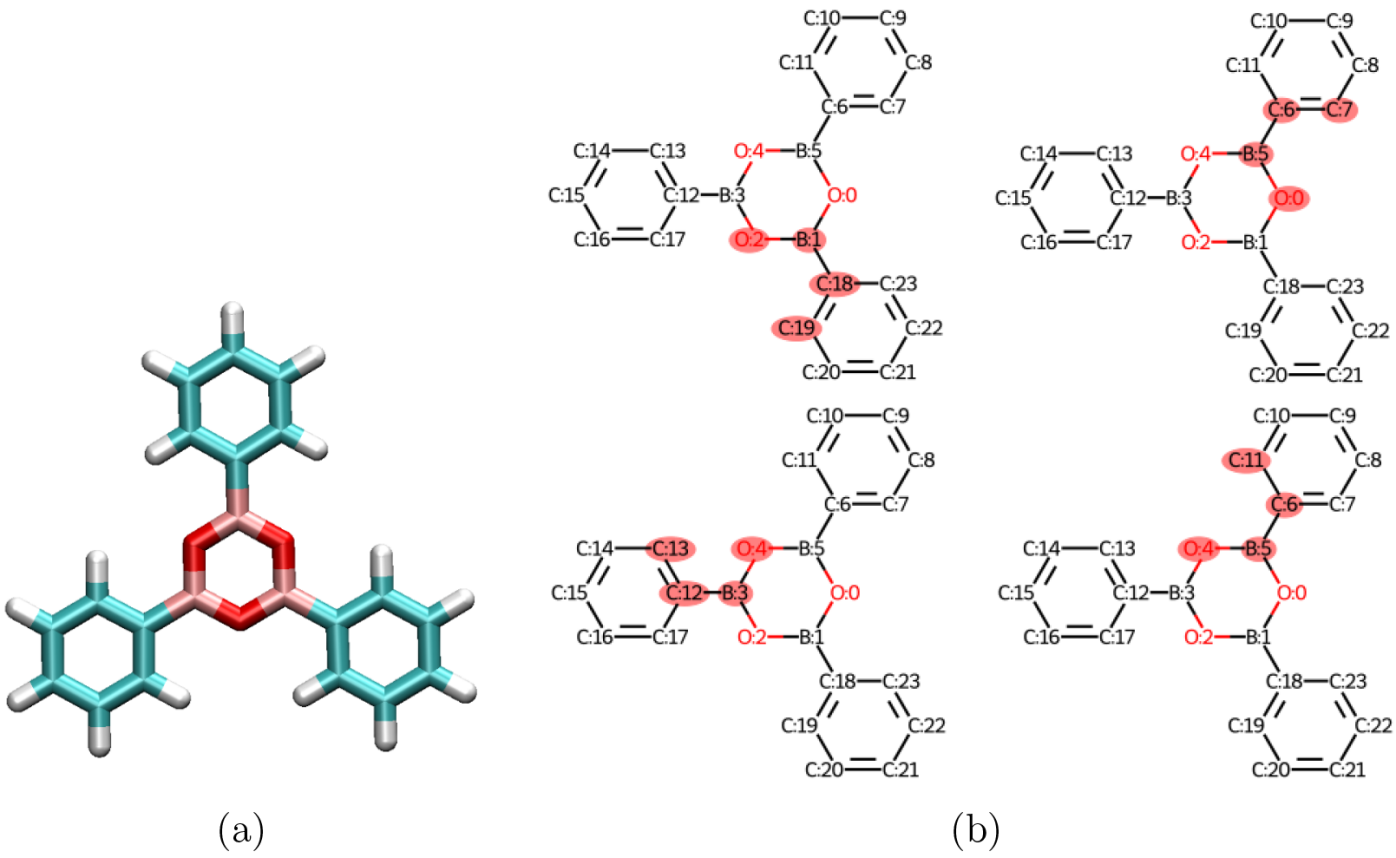
Automatic recognition of intermolecular torsional angles within a planar polymer. (a) Boroxine planar polymer. (b) 2D depiction of selected torsional angles highlighting the atoms and their numerical labels.

This module then reports all of these important torsional angles within the molecule and generates 2D molecular sketches of each one. Figure 15 shows an example of this output for a boroxine polymer.^45^

## Data Provenance and Software Development

**Figure 16:**
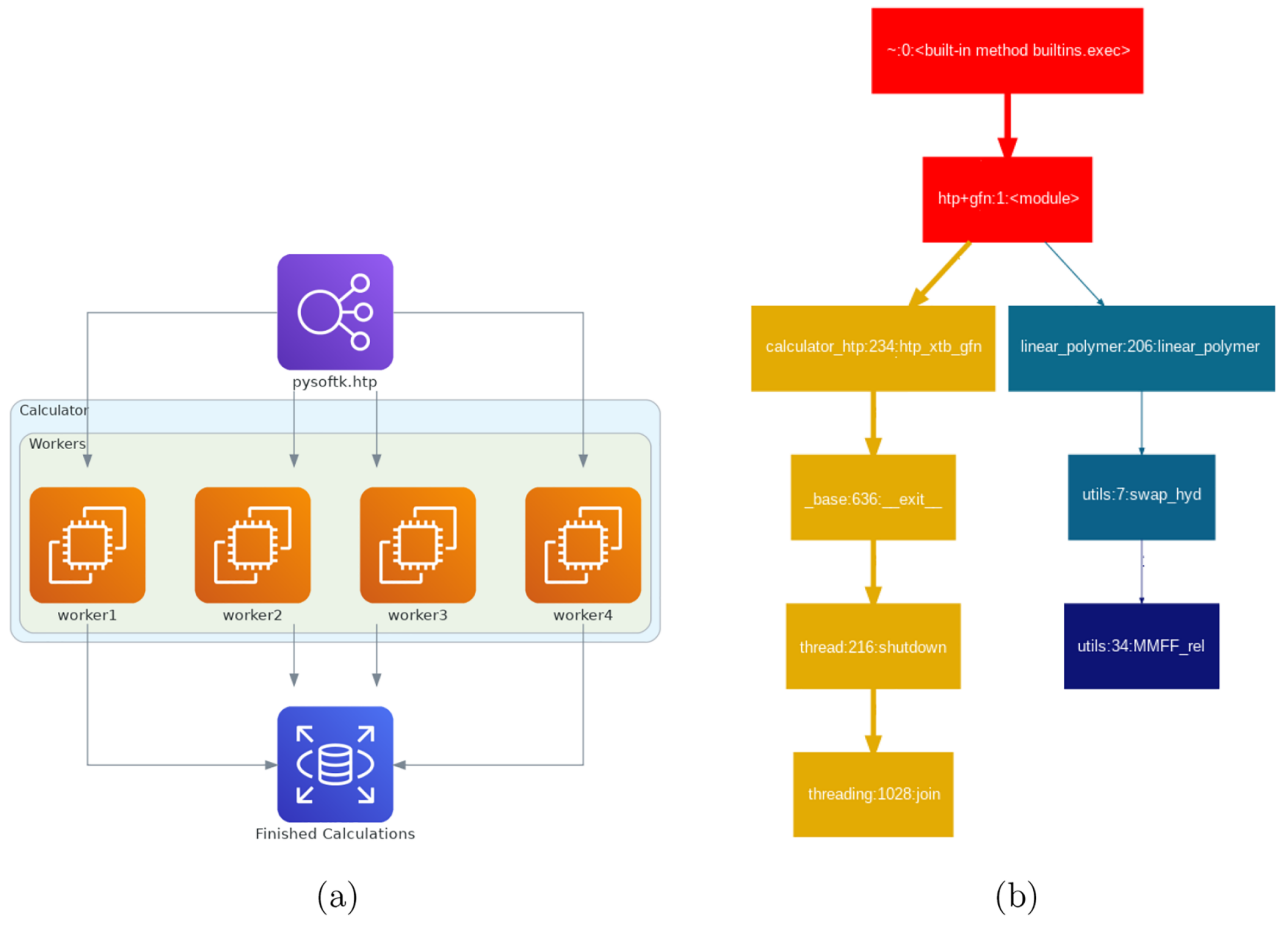
Parallelization strategies implemented in PySoftK. (a) Schematic diagram presenting the distribution of user defined workers (threads). Each worker performs a calculation using different functions of PySoftK (i.e *pysoftk.calculator*, *pysoftk.linear*_*polymer*, *pysoftk.utils.swap*_*hyd*, and *pysoftk.MMFF*_*rel*) object where further parallelization schemes can be used (cores). (b) Usage report of a calculation performed using an Intel(R) Core(TM) i9-900 CPU 3.10 GHz computer employing 16 polymers created on-the-fly in a 4 × 4 scheme where four threads are performing task management while four cores are used to perform semiempirical quantum mechanical calculations at the GFN2-xTB level of theory.

One of our main objectives while creating PySoftK is to provide tools to the community that allow users to utilize high-performance computational facilities to automate the creation of databases for polymeric structures as easily as possible. To do so, we have greatly simplified the installation process of PySoftK by employing the pip command strategy. PySoftK has been tested in Linux and Mac operating systems based on Python 3.6 or more recent versions. Parallel strategies have been developed in parts of the code to enhance the scalability in tasks such as HTC or general organization of molecular databases. In this sense, we have developed modules of PySoftK using concurrency where independently executing tasks are created and queued to use the available resources. PySoftK uses PySCF, a Python-based ab initio computational chemistry program where different theoretical methods (such as Hartree–Fock, MP2, Density Functional Theory, AM1, and MINDO semiempirical methods) are implemented to perform geometry optimizations.^46^ Similarly, the GFN(1,2)-xTB tight-binding semiempirical methods and the associated polarizable classical force-field (GFN-FF) are also enabled.^47^ This approach ensures that the calculator module can run utilizing all available resources, combining task assignment and core usage for parallel codes such as xTB suite, while efficiently avoiding computational bottlenecks. This is depicted schematically in Figure 16.^48^ The user can therefore maximize the parallel performance of the code.

PySoftK has been constructed using principles of modern code development practices. Thus, continuous integration (CI) strategies have been incorporated to probe the code integrity against suggested changes. In our case, these tests have been designed not only as a showcase of all of the capabilities of PySoftK, but also with the aim of maximum code coverage achieved with our current test set. This design ensures that new commits are compatible with the majority of the modules before a new version is integrated into the main branch and released.

Continuous deployment (CD) is achieved once all previous tests have passed, enabling agile updating of PySoftK. At this point, a successful CD is only achieved when the corresponding explanatory examples are included in the documentation. This process is achieved since the documentation is also part of the PySoftK building process. Finally, detailed documentation (which is also part of our CI/CD workflow) has been developed showing working examples (based on our tests) alongside of tutorials for all the different features enabled in PySoftK. They have been tested by members of our community, and feedback has been incorporated into the latest version of the code, ensuring a clear and concise approach for new users.

## Unique Features of PySoftK

Recently several software packages that have some of the same features found within PySoftK have been published including RadonPy,^49^ stk,^50^ Polymer Structure Predictor (PSP),^34^ and the CHARMM-GUI Polymer Builder.^51^ However, PySoftK has several unique features in comparison to these codes. The primary functionalities of these codes are compared in Table 1.

**Table 1:** Comparison of Polymer Model Building Software Packages^a^

		PySoftK	RadonPy	stk	PSP	CHARMM-GUI
**Polymer building**						
*Topology*						
	Linear	Y	Y	Y	Y	Y
	Branched	Y	N	N	N	N
	Ring	Y	N	N	Y	N
*Composition*						
	Homopolymer	Y	Y	Y	Y	Y
	Block copolymers	Y	Y	Y	N	Y
	Alternating	Y	Y	Y	N	Y
	Random	Y	Y	Y	N	Y
						
**High throughput calculations**						
*Polymer generation*		Y	Y	Y	N	N
*Data management tools*		Y	N	Y	N	N
						
**Structure optimization**		Semiempirical or ab initio	ab initio	—	classical FF	classical FF
						
**Simulation cell generation**		N	Y	N	N^b^	Y
						
**Automatic FF assignment**		—	GAFF2	—	OPLS^c^ and GAFF2	CHARMM

a Several key functionalities for generating polymer systems for classical simulations are summarized in this table for PySoftK, RadonPy,^49^ stk,^50^ Polymer Structure Predictor (PSP),^34^ and the CHARMM-GUI Polymer Builder.^51^

b Uses PACKMOL.^52^

c Calls LigParGen^37^ so is restricted to 200 atoms.

There are also other more specific functions that PySoftK has which are not found in other codes, like the tool to identify the important dihedrals that are commonly needed to be reparametrized in conjugated polymers and the ability to uniquely interface it with other programs like Orca^53^ and PySCF.^54^ Meanwhile, RadonPy has an extensive list of tools to perform analysis of the polymers that are simulated within the software package.

## Conclusions

PySoftK is a modular and versatile software that has been designed to facilitate high-throughput calculations of polymeric systems. The code contains a range of modules that allow its users to generate polymers with uniquely broad ranges of topologies and compositions. Additionally, the code has unique tools to assist in the file and directory structure management which is inherent in high-throughput calculations. All of the functionality within PySoftK has been developed to be embedded as part of user-defined workflows enabling steps such as modeling or computing using the specifically defined modules. In a complementary fashion, PySoftK allows the user to keep a track record of the data produced, facilitating postprocessing analysis that can be also part of the same workflow. An ample set of testing scripts has been created aiming at a high coverage of the code which ensures that future algorithm developments are preserving the code structure. Further documentation and tutorials are available on the PySoftK website (https://alejandrosantanabonilla.github.io/pysoftk/). The modular nature of the code allows for the user community to easily contribute code to complements its existing functionality.

## Data Availability

All of the data and scripts necessary to utilize PySoftK are provided within the text of this manuscript and/or within the website for PySoftK (https://alejandrosantanabonilla.github.io/pysoftk/). Within the website of PySoftK, we also have additional tutorials for using the various aspects of the code. The scripts used to generate the various polymers presented in this manuscript can be found on the following website: https://github.com/Lorenz-Lab-KCL/pysoftk_inputs_tests, alongside some tests we have performed of PySoftK. Finally, the code is freely available from the following github page: https://github.com/alejandrosantanabonilla/pysoftk.

## Supporting Information Available

Results of a computational benchmark for testing the Htp module of PySoftK together with the pictures of all bonds detected for boroxine (PDF)
