# Supplementary material for: Modular Software for Generating and Modeling Diverse Polymer Databases
Source: J Chem Inf Model. 2023 Jun 8;63(12):3761–71. doi: 10.1021/acs.jcim.3c00081 (PMC10302471; doi:10.1021/acs.jcim.3c00081)
Supplement: Supplementary file 1 — ci3c00081_si_001.pdf [file ci3c00081_si_001.pdf]

Supporting Information
Modular Software for Generating and Modelling Diverse Polymer Databases

Alejandro Santana-Bonilla,[*,†] Raquel López-Ríos de Castro,[‡,¶] Peike Sun,[¶] Robert M. Ziolek,[¶] and Christian D. Lorenz[*,¶]

†*Department of Physics, King's College London, London, WC2R 2LS, United Kingdom*

‡*Department of Chemistry, King's College London, London, SE1 1DB, United Kingdom*

¶*Biological Physics and Soft Matter Group, Department of Physics, King's College London, London, WC2R 2LS, United Kingdom*

E-mail: alejandro.santana_bonilla@kcl.ac.uk; chris.lorenz@kcl.ac.uk

Benchmark result for asynchronous parallelization

PySoftK has been developed to support the production of high-throughput calculations. To this end, the asynchronous parallelism model has been chosen to enable the creation of workflows that combines polymer modelling and calculations at different levels of theory. Tests have been performed using the script displayed in Figure S1.

Using gprof2dot[1] for profiling PySoftK on an Intel(R) Core(TM) i9-900 CPU 3.10 GHz computer, we tracked different possible configurations for a set of 10 polymers which were increasing in length, and the results are shown in Figures S2 and S3. This is representative of a small series of high-throughput calculations for heterogeneous systems like polymers.

```python

from rdkit import Chem
from rdkit.Chem import AllChem

from pysoftk.linear_polymer.super_monomer import *
from pysoftk.linear_polymer.linear_polymer import *
from pysoftk.format_printers.format_mol import *
from pysoftk.folder_manager.folder_creator import *
from pysoftk.htp_tools.calculator_htp import *

mol_1=Chem.MolFromSmiles('c1cc(sc1Br)Br')
mol_2=Chem.MolFromSmiles('c1cc(sc1Br)Br')
=Sm(mol_1,mol_2,"Br")

molecules=[]
for i in range(1,10):
   k=a.mon_to_poly()
   molecules.append(Lp(k,"Br",i,shift=1.0).linear_polymer("MMFF",150*i))

for idx, values in enumerate(molecules):
  Fmt(values).xyz_print("monomer_"+str(idx)+".xyz")

Fld().file_to_dir("xyz")

# High-throughput calculations at the gfn-ff level of theory
Htp("xyz").htp_xtb_gfn("xtb",4,1)
```

Figure S1: Code snippet showing the benchmark procedure using PySoftK Htp module.

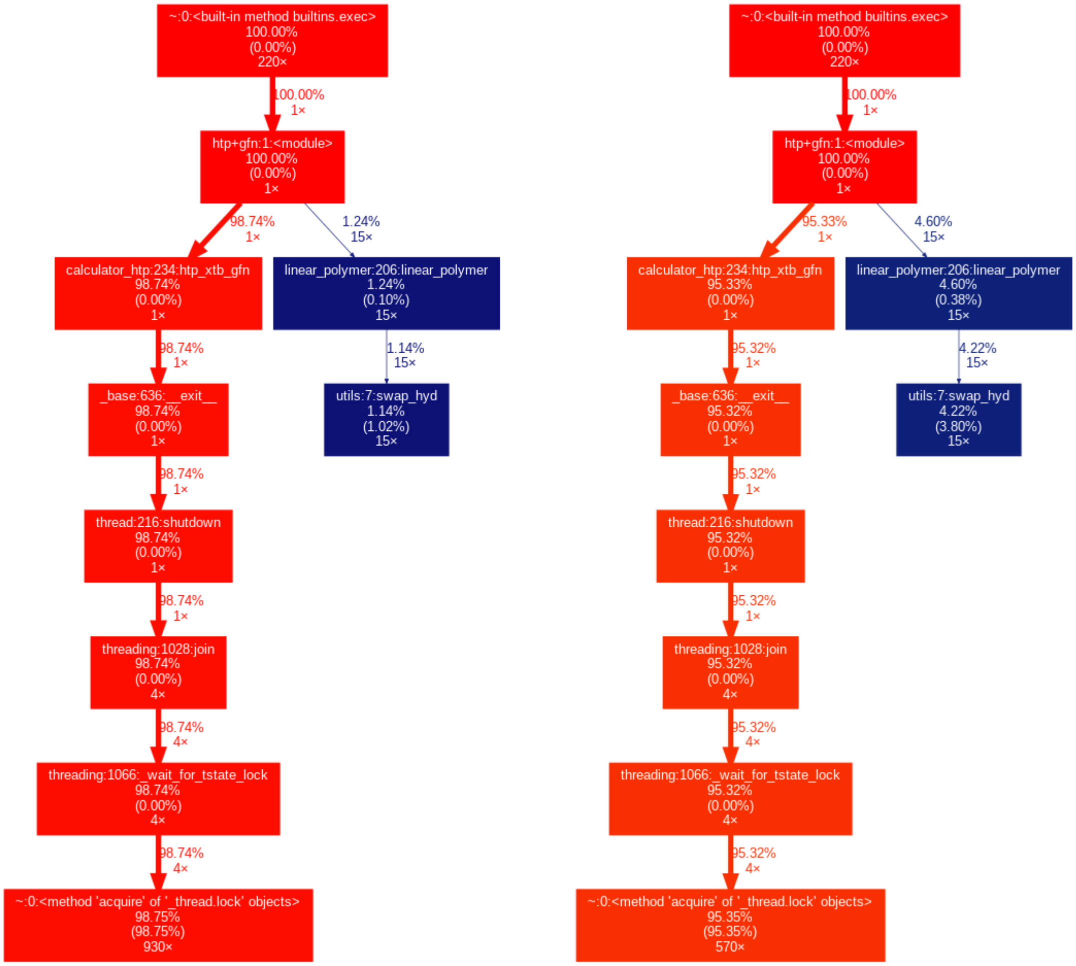

(a) (b)

Figure S2: Benchmark analysis for different combination at different level of parallelism. PySoftK controls the number of calculations submitted whereas calculators such as GFN2-xTB and PySCF employ the rest of available resources. (a) **PySoftK** 1 thread **GFN2-xTB** 1 core. (b) **PySoftK** 1 thread **GFN2-xTB** 4 cores.[2]

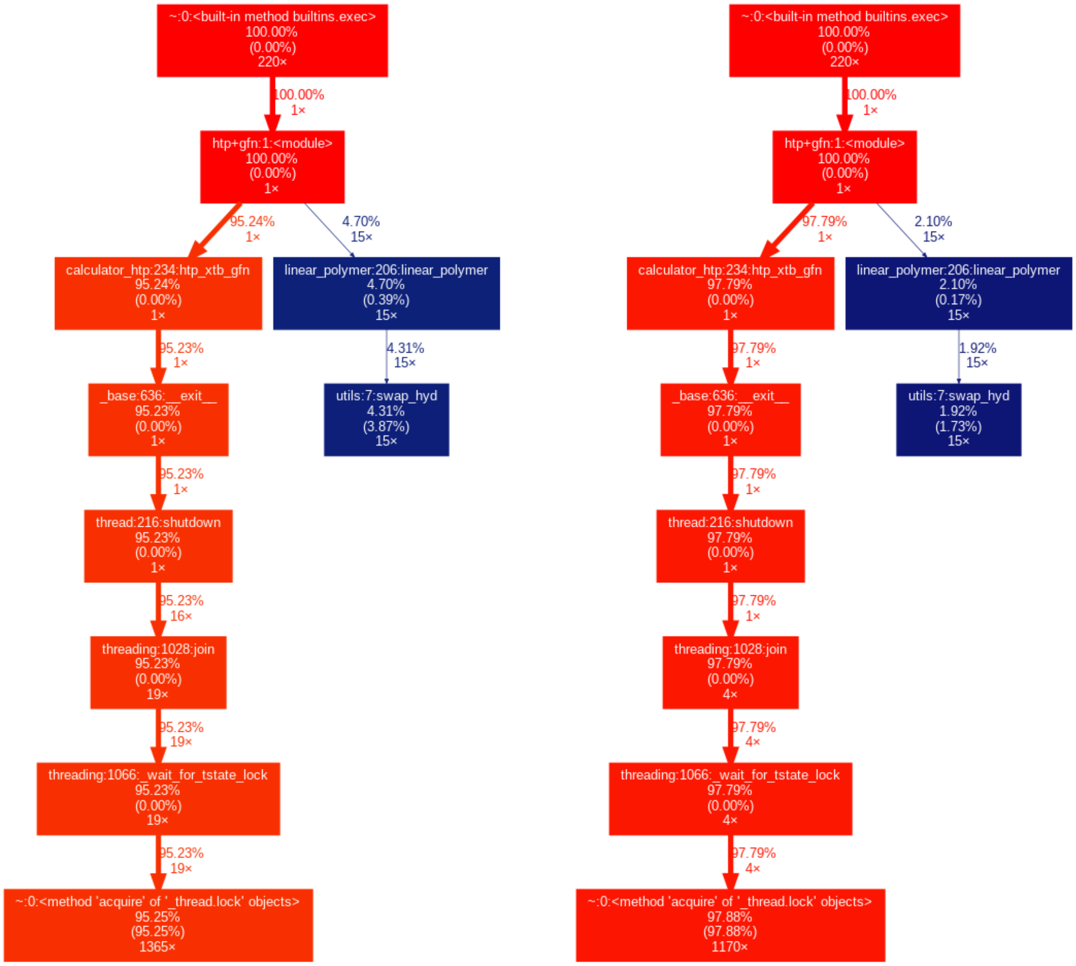

(a) (b)

Figure S3: Benchmark analysis for different combination at different level of parallelism using GFN2-xTB as calculator. (a) **PySoftK** 16 threads **GFN2-xTB** 1 core. (b) **PySoftK** 1 threads **GFN2-xTB** 16 cores.[2]

Torsional angles for boroxine.

In this section, we display all of the different detected angles for boroxine, which is displayed in Figure 15. The results are presented in Figures S4 and S5 and the code to obtain these results is displayed in Figure S6.

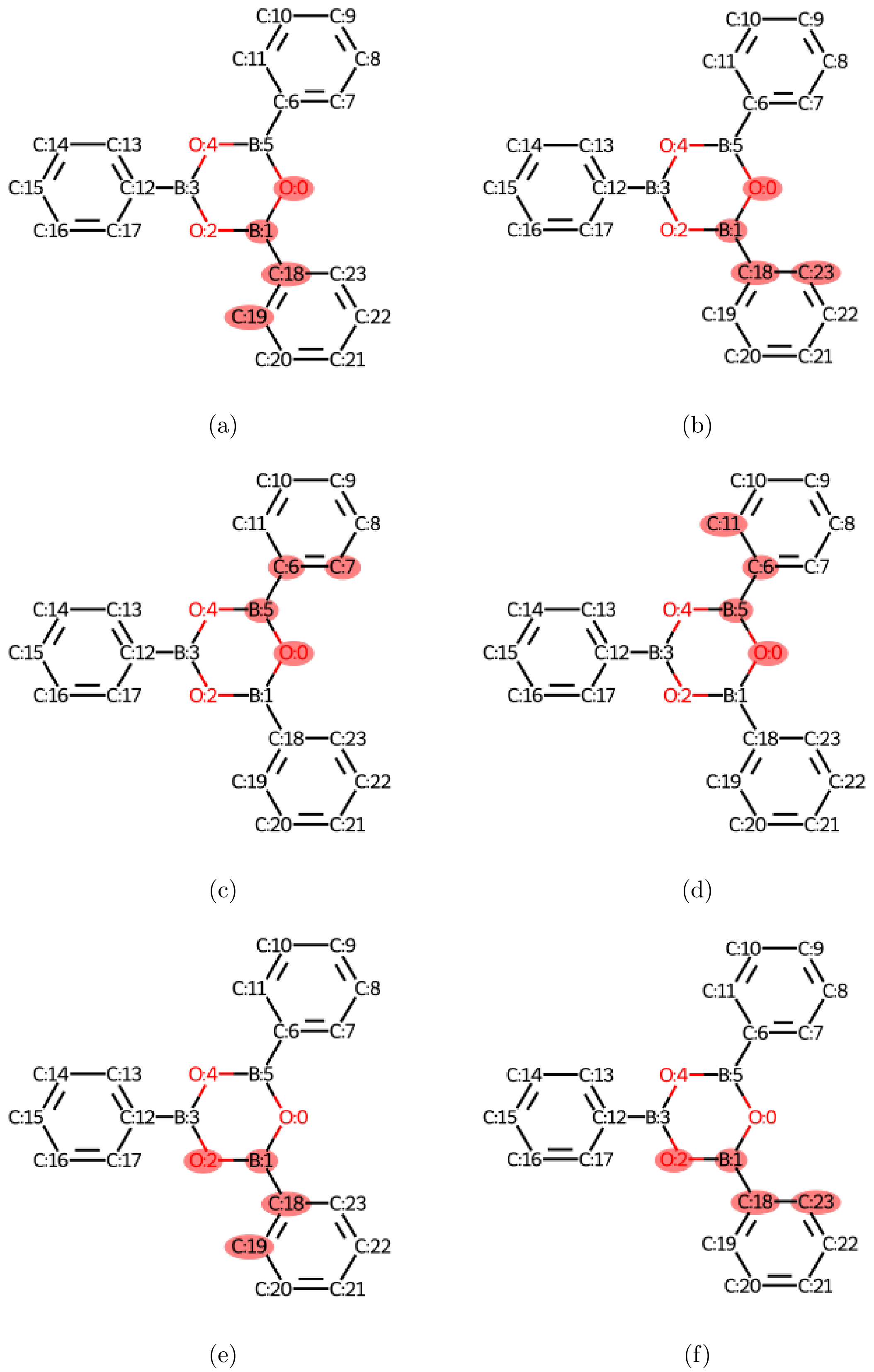

Figure S4: First set of results for automatic torsional angle detection for boroxine.

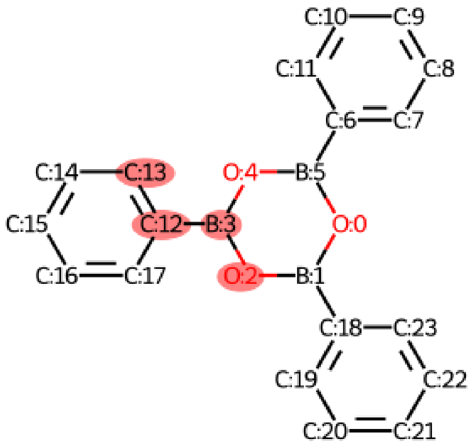

(a)

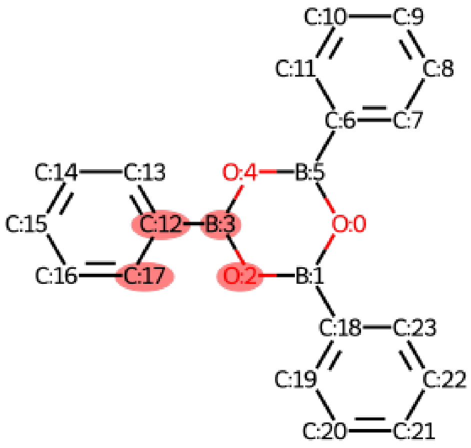

(b)

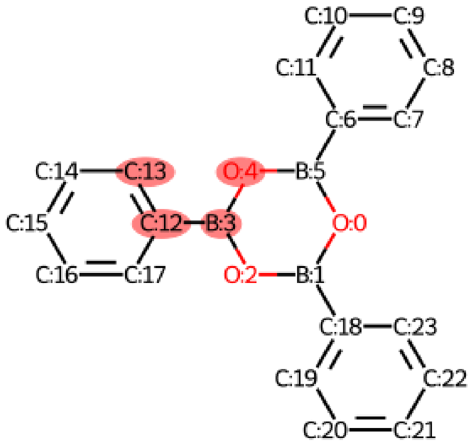

(c)

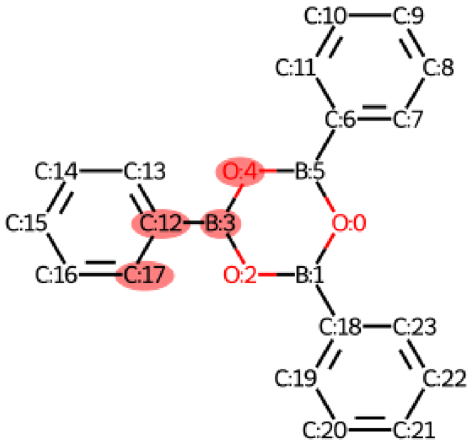

(d)

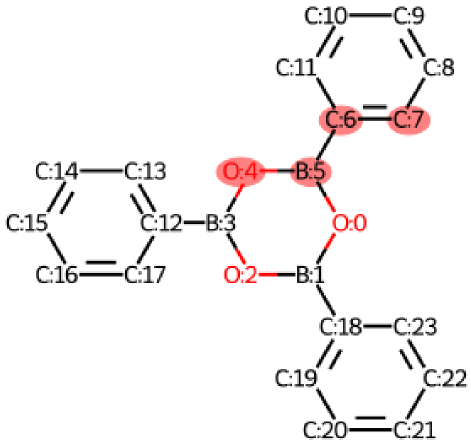

(e)

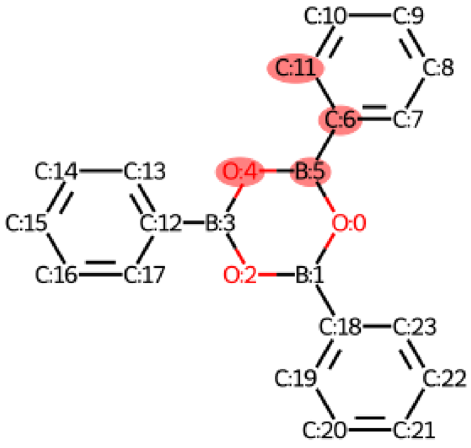

(f)

Figure S5: Second set of results for automatic torsional angle detection for the molecule Boroxine.

```
from rdkit import Chem
from rdkit.Chem import AllChem

from pysoftk.torsional.torsional import *

#Test counting the torsional angles in a planar polymer
molecules=Chem.MolFromSmiles(’O1B(OB(OB1c1ccccc1)c1ccccc1)c1ccccc1’)
Torsional(molecules).plot_trs_ang("mol_1")
```

Figure S6: Code snippet showing the Torsional angle code using the corresponding PySoftK module.

References

(1) Fonseca, J. gprof2dot. https://github.com/jrfonseca/gprof2dot, 2022; accessed 2022-10-01.

(2) Bannwarth, C.; Ehlert, S.; Grimme, S. GFN2-xTB—An accurate and broadly parametrized self-consistent tight-binding quantum chemical method with multipole electrostatics and density-dependent dispersion contributions. *J. Chem. Theory Comput.* **2019**, *15*, 1652–1671.
